# Dispersal limitation of *Tillandsia* species correlates with rain and host structure in a central Mexican tropical dry forest

**DOI:** 10.1371/journal.pone.0171614

**Published:** 2017-02-03

**Authors:** Elizabeth Victoriano-Romero, Susana Valencia-Díaz, Víctor Hugo Toledo-Hernández, Alejandro Flores-Palacios

**Affiliations:** 1 Centro de Investigación en Biodiversidad y Conservación (CIByC), Universidad Autónoma del Estado de Morelos, Av. Universidad, Col. Chamilpa, Cuernavaca, Morelos, México; 2 Centro de Investigación en Biotecnología (CEIB), Universidad Autónoma del Estado de Morelos, Av. Universidad, Col. Chamilpa, Cuernavaca, Morelos, México; Chinese Academy of Forestry, CHINA

## Abstract

Seed dispersal permits the colonization of favorable habitats and generation of new populations, facilitating escape from habitats that are in decline. There is little experimental evidence of the factors that limit epiphyte dispersion towards their hosts. In a tropical dry forest in central Mexico, we monitored the phenology of dispersion of epiphyte species of the genus *Tillandsia*; we tested experimentally whether precipitation could cause failures in seed dispersal and whether seed capture differs among vertical strata and between host species with high (*Bursera copallifera*) and low (*Conzattia multiflora*) epiphyte loads. With the exception of one species that presents late dispersion and low abundance, all of the species disperse prior to the onset of the rainy season. However, early rains immobilize the seeds, affecting up to 24% of the fruits in species with late dispersion. We observed that *Tillandsia* seeds reach both *Bursera* and *Conzattia* hosts, but found that adherence to the host is 4–5 times higher in *Bursera*. Furthermore, seeds liberated from *Bursera* travel shorter distances and up to half may remain within the same crown, while the highest seed capture takes place in the upper strata of the trees. We conclude that dispersion of *Tillandsia* seeds is limited by early rains and by the capture of seeds within the trees where populations concentrate. This pattern of capture also helps to explain the high concentrations of epiphytes in certain hosts, while trees with few epiphytes can be simultaneously considered deficient receivers and efficient exporters of seeds.

## Introduction

Dispersion is the unidirectional movement of an individual away from its site of origin [1] and is a process that enables colonization of new sites and influences genetic flow [1, 2]. The predominance of a given dispersion type among different groups of plants depends on both the guild studied and the environment. For example, anemochory is common in habitats with low and seasonal precipitation (*e*.*g*. dry climates) [3] and in epiphytic plants [4].

Epiphytes comprise approximately 9% of the vascular plant species worldwide [5] and it is estimated that 84% of the epiphytes have anemochorous diaspores, which could represent a strategy for colonizing the crowns of trees [6]. One of the main groups of epiphytes is the Bromeliaceae family, which comprises at least 1800 epiphytic species [5]. Among the bromeliads, seeds of species of the subfamily *Tillandsioideae* have comose appendages that assist dispersion by wind, facilitate adherence of the seed to tree bark, fix the seedling in place until development of the roots and intervene during germination, absorbing and retaining solutions with which the seedling comes in contact [7, 8, 9, 10].

Epiphyte species can have distributions that show bias towards some host species and vertical strata [10, 11, 12]. Species distribution is determined in part by the availability of seed sources, but even where sufficient seeds are produced, failure to disperse these in sufficient quantity in the landscape is known as “dispersal limitation” [13, 14].

There has been little study of epiphytic bromeliad seed dispersion [15, 16, 17]; however, it has been observed experimentally that most *Tillandsia* seeds disperse over distances of less than 20 m [15, 16]. It has also been suggested that dispersion of *Tillandsia* seeds of the tropical dry forest is limited by the rain that hydrates the comose appendages of the seed, creating clumps of immobilized seeds on the inflorescence itself [16] [S1 Fig]. It is expected that the opening of *Tillandsia* capsules is concentrated towards the end of the dry season, when the rains are infrequent and of low intensity, maximum air temperatures occur, diaspores become dry, seeds complete their maturation and are ready to germinate when the rains begin [18]; however, there are no data to confirm if this is indeed the case for the vascular epiphytes.

It is possible that the differential distribution of epiphytes among tree species and strata may be influenced by the range of capacities of the seeds to adhere to different bark that occurs among trees species and strata. Each tree species have specific bark traits (e.g. texture, peeling rate) but these differences also exist within each tree; while the bark of a twig is usually smooth, the bark of older areas (e.g. trunks) could be rugose and peeling rates could differ among strata [19]. In the vertical strata [20], branches and exterior twigs are more numerous and could form “nets” that intercept the seeds [10, 21] that originate from epiphytes growing on either the same or different trees, thus acting to limit emigration of seeds from trees with high epiphyte loads [10].

In the tropical dry forest of central Mexico, *Bursera bipinnata*, *B*. *copallifera* and *B*. *glabrifolia* concentrate individuals of *Tillandsia*; while taller trees such as *Conzattia multiflora* support a lower number of individuals than would be expected given their abundance [11]. Epiphytes are stratified vertically on their hosts; for example, on *B*. *copallifera* and *B*. *glabrifolia* (both with a non-peeling bark), they are concentrated on exterior branches of between 2 and 4 cm in diameter (zone IV of Johansson), but are scarce on the trunks and on twigs (<2 cm). In *C*. *multiflora*, however, epiphytes are more frequent on the trunks [10].

In this study, we monitored the phenology of dispersion of five *Tillandsia* species; we tested experimentally the effect of precipitation as a cause of failure in seed dispersal, and whether seed capture differs among vertical strata and between host species with high (*Bursera copallifera*) and low (*Conzattia multiflora*) epiphyte loads, and the distance that the seeds disperse. We hypothesized that: a) seed dispersion is concentrated at the end of the dry season, b) the sporadic and low intensity rains that occur in the dry season cause immobilization of seeds, c) more seeds adhere to branches of tree species with a greater abundance of epiphytes (“preferred”) than to those of tree species with few epiphytes (“limiting”) and d) highest seed capture occurs in the exterior strata of the canopy which is where a higher number of structures (*e*.*g*. branches and twigs) [10, 21] are present.

## Methods

### Study area

This study was carried out in the tropical dry forest of Tenextepec hill (Cerro de la Cal) in San Andres de la Cal, Tepoztlán, in Morelos, Mexico (18°57’26.73” W, 99°06’24.87” N; 1495 m asl). This area belongs to the people of San Andrés de la Cal, represented by the President of the communal land who granted us permission to carry out this survey. Mean annual temperature at the study site is 20.5°C and mean annual precipitation is 1091.8 mm [11]. The rainy season is restricted to the period between May and October. During the dry season, the winds are mainly easterly, while during the rainy season the winds are northerly or southerly [22].

Luvic Phaeozem soils cover the hills of San Andres of the Cal [22], where at least 42 woody species (DBH >3 cm) have been recorded. The most abundant of these are *Sapium macrocarpum* Müll. Arg. (Euphorbiaceae), *Bursera fagaroides* (Kunth) Engl., *B*. *glabrifolia* (Kunth) Engl. (Burseraceae), *Ipomoea pauciflora* M. Martens & Galeotti, *I*. *murucoides* Roem & Schult (Convolvulaceae) and *Conzattia multiflora* (B.L. Rob.) Standl. (Fabaceae) [11]. The epiphytic flora consists of 19 species of which 53% are true epiphytes while the rest are accidental [11]. One of the most abundant epiphytes is *Tillandsia recurvata* (L.) L.; individuals of this species account for 72% of the epiphytes present in the area. Individuals of *T*. *hubertiana* Matuda comprise 2.5% of the true epiphytes in the hills of San Andres de la Cal [11], but this is the species that produces most seeds per fruit and most fruits per inflorescence [23, 24] Further details of the study zone and about the reproductive biology of the *Tillandsia* species can be found in [11, 23, 24].

### Phenology of capsule opening

We followed the phenology of seed capsule opening from February to May-July in two different years (2010–2011). Previous observations in the same study area [24] showed that fruit opening occurs toward the end of the dry season (April-May); however, in the first season, we followed the opening of capsules until July, because some capsules remained closed, while in the second season we only followed the opening of capsules until May, since all of the capsules had opened by then. In the first period (February 3rd—July 28th, 2010), on a transect of 10 × 100 m (0.1 ha), all capsules of any *Tillandsia* species of up to 2 m in height were marked [total = 174 capsules; 16 capsules of *T*. *achyrostachis* (4 individuals with 1–8 capsules), four of *T*. *caput-medusae* (1 individual), 146 of *T*. *recurvata* (42 individuals of 1–12 capsules) and eight of *T*. *schiedeana* (1 individuals)]. The branch where each capsule was found was marked with tape (Wood Fiber Enviro-Flagging 1136, Forestry Suppliers Inc., USA). Every week, we checked the capsules and quantified any dehiscence.

Due to the low number of marked capsules of species other than *T*. *recurvata* in the 2010 period, a higher number of capsules of *T*. *achyrostachis* (106 capsules, 37 individuals with 1–6 capsules), *T*. *caput-medusae* (109 capsules, 9 individuals with 3–33 capsules), *T*. *circinnatioides* (50 capsules in 10 individuals with 2–14 capsules), *T*. *recurvata* (100 capsules, in 19 individuals with 2–21 capsules) and *T*. *schiedeana* (107 capsules, in 13 individuals with 1–22 capsules) were marked in February 2011 and monitored from February 17th to May 19th. For this purpose, we searched outside of the 10 x 100 m transect until obtaining a minimum of 100 capsules per species; the exception was *T*. *circinnatioides* which is one of the less abundant species in the study area (0.2%) [11]. The capsules were monitored weekly over the 2011 periods of dispersion (472 capsules in total).

### Effect of rain on seed dispersion

In order to determine whether rain is a factor that immobilizes seeds and prevents their dispersion, before the capsules opened in February 2011, 10 marked capsules of each *Tillandsia* species (4 capsules for *Tillandsia circinnatioides*) were isolated from the rain inside plastic bags and, with a manual spray, moistened weekly with 4 ml of water (Sprayed treatment). The same numbers of capsules per species were also isolated from the rain in plastic bags (Bagged treatment) but were not moistened; while the remaining capsules were left exposed to the rain (Open treatment). These treatments were applied to all of the species with marked capsules during the 2011 season. At the beginning of the rainy season (mid May, when the first rain felt), capsules were collected and any seeds that were immobilized and still adhered to the capsules/inflorescences were quantified; it was not possible to count non-adhered seeds because these had dispersed in the Open treatment. An immobilized seed was defined as a seed that had lost its capacity to fly because its appendages had become joined together, and were fixed to the fruit or to the inflorescence from which the seed originated [S1 Fig].

### Seed capture and dispersion distance between hosts and strata

For this experiment, we selected the focal tree species *Bursera copallifera* and *Conzattia multiflora*. Both species are representative of conserved forest in the study area [25] and both have a non-peeling bark [11]. *Bursera copallifera* is a maximum of 6–7 m in height, its bark is rugose and its crown is dense and supports a high load of vascular epiphytes. *Conzattia multiflora* reaches 10–14 m in height, its bark is smooth and its crown is open with a low density of branches and a low load of epiphytes [10, 11].

In order to determine the success of seed dispersion among *C*. *multiflora* and *B*. *copallifera* and their strata, two pairs of trees were selected as seed sources. Each pair consisted of one individual each of *C*. *multiflora* and *B*. *copallifera*, the first pair were located 3 m apart and the second 6 m apart; while the distance between the two pairs was 22–25 m. With this design, it was possible to measure the quantity of seeds that dispersed from a preferred (*B*. *copallifera)* and from a limiting (*C*. *multiflora*) tree, and remained on the same tree (trapped), as well as those that reached neighboring trees and the distance they travelled. All trees within a 15 m radius around each member of the two pairs of trees were marked. All trees were georeferenced (Garmin, GPSMAP-60, USA) at their base, in order to establish the distances between them.

In the trees of each pair, 10,000 seeds were released from the branches of the exterior stratum. For this release, one metal strainer (25 cm in diameter) was placed on the inner branches oriented towards each of the four cardinal points (North, South, East and West) and another was placed in the center of the crown. Each of the total of five strainers was filled with 1000 seeds which were then left to disperse naturally through the actions of air movement and gravity. This assay was conducted on two occasions per each tree (2 tree individuals x 2 tree species = 4 trees), allowing a week to elapse between each assay per tree (2 tree individuals x 2 tree species x 2 moments or seed release = 8 assays). The dispersion experiment was conducted during the peak of capsule dehiscence (April). The seeds used in each assay were of *T*. *recurvata* (2000) and *T*. *hubertiana* (3000 seeds). These were mixed and marked with fluorescent powder (BioQuip Products Inc., #1162B Luminous powder-blue, #1162R Luminous powder-red, #1162Y Luminous powder-yellow, #1162W Luminous powder-white, USA). For powder marking, the seeds of each assay were separated into glass jars, dried at 30°C for 48 hours in an oven (Binder, model FD 115-UL, USA) and then dyed with the powder. The seeds were kept dry until placed in each strainer. In order to recognize the seeds used in each assay, unique colors were used for each assay, either one of the original powder colors (blue, red, yellow and white) or one created by combining two powder colors (pink, purple, orange and green).

In order to determine the arrival of the painted seeds to different trees and strata, the day after their release from the strainers, we searched freely around the trees and between both tree couples. During the dry season the TDF is empty of leaves and any colored object is visually striking, so in the surroundings of the focal trees we looked for seed clumps adhered to the vegetation, on the forest floor or in the seed traps. Seed traps were placed in the trees used as seed sources (4 trees) and each of the 27 trees located within the 15 m radius (31 trees in total). The 372 seed traps consisted of plastic strips (2 cm x 30 cm = 0.006 m^2^) covered with an insect trapping resin (Tanglefoot Company, Product 99080, USA). Traps were tightly attached to the bark surface of the trees with cable ties (Steren) [S2 Fig]. On each tree, one seed trap was placed in each vertical stratum (of three strata) and at each cardinal orientation within each stratum (North, South, East and West; three strata x 4 orientations = 12 traps per tree). Zones I and II as described by [20], were considered as one zone, given that the trees in the tropical dry forest are small (5–12 meters) and have short trunks that bifurcate within the first few meters [10]. Zones III and IV (interior) were also considered together, since some tree species have crowns of reduced size. Finally, the zones of the trunk (I-II) and the interior (III-IV) and exterior (zone V) branches were used as vertical strata. The trees where traps where placed were of the species *Bursera copallifera* (9), *Cedrela oaxacensis* C. DC. & Rose (Meliaceae) (1), *Conzattia multiflora* (5), *Cordia morelosana* Standl. (Boraginaceae) (1), *Gliricidia sepium* (Jacq.) Standl. (Fabaceae) (1), *Ipomoea pauciflora* Mart. & Galeotti (Convolvulaceae) (11), *Mastichodendrom capiri* (A.D.C) (Sapotaceae) (1) and *Spondias purpurea* L. (Anacardiaceae) (2).

### Seed adherence to host branches

In order to determine whether seed adherence to the branches differed among host species, an experiment was conducted during the dispersion period of the year 2010. For this experiment, seed lots of *T*. *hubertiana* and *T*. *recurvata* were blown over branches of *B*. *copallifera* and *C*. *multiflora* through a glass duct [S3 Fig]. The duct consisted of a steel structure with glass walls. A fan with a strainer on top was placed at the base of the duct. The seeds used in each assay were placed in the strainer. In each assay, we fixed six branches (3 of *B*. *copallifera* and 3 of *C*. *multiflora*) in a random order to the upper opening of the duct, at a distance of 0.9 m from the seeds. The six branches used in each assay were from different individual trees and were fixed parallel to each other and each branch was used in only one assay. The branch diameters used in the assays did not differ *(t* = 1.0, *d*.*f*. = 92, *P* = 0.32) between *B*. *copallifera* (10.4 ± 3.5 mm; hereafter mean ± SD) and *C*. *multiflora* (11.1 ± 2.7 mm). Ten assays were conducted per *Tillandsia* species, with each assay utilizing 100 seeds. At the end of each assay, the number of seeds that had adhered to each branch was recorded, as well as those that remained in the duct and on the strainer.

Wind speed and relative humidity were measured in a non-forested zone adjacent to the studied forest, on the 21st (9:45–11:10 hrs.), 23rd (11:20–13:00 hrs.), 24th (8:30–17:20 hrs.) and 25th (9:30–19:20 hrs.) of April 2010. These measurements were conducted every ten minutes with an anemometer (EXTECH Instruments, model 45158 Mini Thermo-Anemometer, USA). Each measurement recorded the average wind speed (one measurement every second), maximum speed reached over a 1-minute period of measurement and average relative humidity (one measurement every 15 s). Average wind speed was 1.6 ± 1.4 m/s, average maximum wind speed was 3.9 ± 2.2 m/s and average relative humidity was 31.4 ± 5.3%. These measurements were taken in the same manner during each assay in the glass duct. Relative humidity did not differ (one-way ANOVA, *F* = 0.05, *d*.*f*. = 2, 89, *P* = 0.96) between the study zone and the laboratory during the assays with seeds of *T*. *hubertiana* (31.6 ± 1.8%) and *T*. *recurvata* (31.7 ± 1.7%). In each assay, the seeds were blown onto the branches at 3.8 ± 0.8 m/s, which did not differ from the average maximum wind speed measured in the study zone over the same period (One way ANOVA, *F* = 0.30, *d*.*f*. = 2, 90, *P* = 0.81).

### Data analysis

#### Phenology of capsule opening

For this analysis, a graph was generated with the accumulated percentages of open capsules per week over each dispersion season (2010 and 2011). Capsules bagged in the experiments were excluded from this analysis. In order to compare graphs between seasons, these were aligned according to the first observation of open capsules. In this way, the speed of capsule opening was compared independently of the time at which each curve begins in each year. In order to test whether there were differences in the speed of capsule opening between years, and between species of *Tillandsia* in the 2011 season, the curves were analyzed with log-rank tests [26]. When significant differences were found, the curves that differed according to Gehan-Wilcoxon paired comparisons were isolated [26].

#### Effect of rain on seed dispersion

In order to compare the number of immobilized seeds between the capsules that were moistened weekly (Sprayed treatment) and those that were untreated (Open treatment), Wilcoxon tests were conducted [27]. The capsules of seeds that were in bags but not moistened (Bagged treatment) were not included in the statistical analysis since they contained no immobilized seeds. While seeds remained inside the bag, their appendages were free, suggesting that the seeds would still have been capable of flight and the seeds were not fixed to either the inflorescence or the fruit. We did not observe capsules with fungus or any pathogens among those that were bagged. Moreover, the percentage of capsules with immobilized seeds was compared between capsules in the Sprayed and Open treatments. This procedure used the method of comparison of two proportions [28], which is based on the z statistic.

#### Seed capture and dispersion distance between hosts and strata

Due to the low arrival of marked seeds, we decided to use also unmarked seeds found in the traps. The sum of both types of seeds was therefore used in the analysis. A chi square (χ^2^) test was used [27] in order to determine whether the quantity of seeds that arrived differed between the strata of *B*. *copallifera* and *C*. *multiflora*. This analysis used the sum of the seeds found in all of the traps in each stratum per tree species. Furthermore, we used a χ^2^ test to check for differences among more than two proportions [28] in order to compare whether the probability of receiving at least one seed differed among strata in *B*. *copallifera*. With the method of comparison of two proportions [28], we compared whether the probability of receiving at least one seed differed among the upper strata of *C*. *multiflora*, since no seed capture occurred on the trunk. In order to increase the number of trees observed, we integrated the *B*. *copallifera* and *C*. *multiflora* individuals that surrounded the focal trees.

In order to test whether the quality of the host determines the quantity of seeds that arrive naturally, we compared the total number of unmarked seeds captured between the traps of *B*. *copallifera* and *C*. *multiflora*. Due to the large quantity of traps that contained no seeds, the total number of natural seeds in the 12 traps (4 by orientation and 3 strata) of each tree was used as a response variable. With a *t*-test for independent data [28], we compared the number of seeds that arrived to the focal trees of *B*. *copallifera* (n = 9) and *C*. *multiflora* (n = 5) and those that arrived to the surrounding trees.

With a χ^2^ test [27], we compared the quantity of marked seeds captured by traps in the branches of the source tree, between preferred and limiting hosts. We compared the quantity of captured seeds between *B*. *copallifera* and *C*. *multiflora* with a Mann-Whitney test for non-independent data [27]. In this test, the pairs of data were each pair of trees (2 tree pairs x 2 moments or seed release = 4 pairs of data) at each time that seeds were released (blue-yellow, purple-orange, white-red and green-pink).

In order to measure the effect of distance on the number of seeds dispersed, an analysis of covariance was conducted with a generalized linear model [29]. This analysis used the number of seeds collected in different traps or sites as a response variable, the distance travelled by the seed as an independent variable and the identity of each assay (random factor, four levels per tree species) and of each tree species (fixed factor, two levels) as factors. Given the fact that the response variable is a count, the model was constructed based on a Poisson distribution [29].

#### Seed adherence to host branches

We used a *t*-test for paired data [28] to determine whether the quantity of captured seeds differed between the branches of *B*. *copallifera* and *C*. *multiflora*. This analysis was conducted for each of the *Tillandsia* species used in the experiment, in which the response variable was the average number of seeds captured by the three branches of each species per assay. All data analyses were performed using Stata 13.1.

## Results

### Phenology of capsule opening

**Fig 1:**
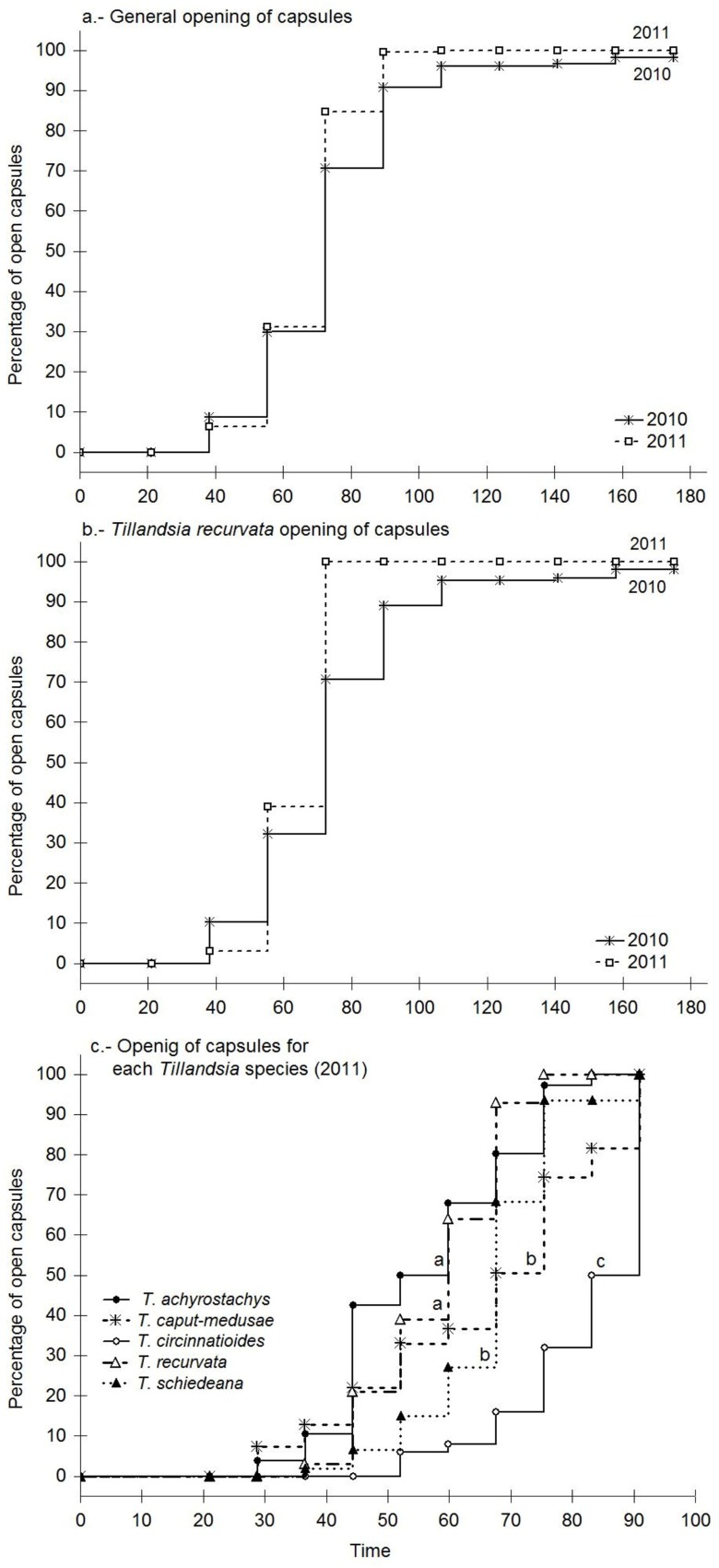
Cumulative number of *Tillandsia* capsules that opened during the dispersion period of 2010 and 2011 in a tropical dry forest in central Mexico. For the 2010 observations, the first opening of capsules occurred on February 24^th^ and for the 2011 period, opening began on February 16^th^. In order to compare between years, the curves were aligned according to the first opening event. Different letters denote significant differences between the curves inside each graph (paired Gehan tests, P<0.05).

In both years, dispersion of the seeds from the monitored capsules occurred between February and April (Fig 1). Capsule opening curves differed between the two years, both in the general curves (log rank χ^2^ = 2.9, *P* < 0.05; Fig 1A) and in the comparison of capsules of *T*. *recurvata* (log rank, χ^2^ = 4.7, *P* < 0.0001; Fig 1B). Maximum opening of capsules in season 2011 was reached more rapidly than in 2010. Capsule opening never reached 100% in 2010, both in the comparison using all species (Fig 1A and 1B) and in the comparison of *T*. *recurvata* between years (Fig 1A and 1B). The reduced capsule opening recorded in 2010 was due to the fact that 3.5% of the *T*. *recurvata* capsules suffered damage through herbivory and consequently failed to open.

Capsule opening curves differed between species (log rank, χ^2^ = 115.0, *P* < 0.0001; Fig 1C). The species that presented fastest opening were *T*. *achyrostachys* and *T*. *recurvata*. These species did not differ between themselves but did with the other species; *T*. *caput-medusae* and *T*. *schiedeana* did not differ between themselves, but did with the other species, and presented intermediate capsule opening speeds, while *T*. *circinnatioides* was the species with the slowest capsule opening (Fig 1C).

### Effect of rain on seed dispersion

In all of the species, there were no immobilized seeds when the capsules were permanently isolated from water (Bagged treatment, Table 1). However, immobilized seeds were found when the capsules were moistened with water (Sprayed treatment, Table 1) and when the fruits were left freely exposed (Open treatment, Table 1). With the exception of *Tillandsia circinnatioides* (the species with the slowest capsule opening), the highest number of immobilized seeds and the highest percentage of fruits with immobilized seeds occurred in all of the species in the Sprayed treatment (Table 1). During the course of this experiment, 10 different rain events occurred (three in April and seven in May), with an average precipitation of 8.7 ± 8.6 mm (minimum = 0.5, maximum 25 mm; Comision Nacional del Agua, unpublished data).

**Table 1:** Percentage of *Tillandsia* capsules with immobilized seeds and number of immobilized seeds attached to the capsules/inflorescence. Capsules were experimentally bagged in order to avoid the rain but treated with sprayed water (Sprayed treatment), bagged to avoid the rain and not sprayed with water (Bagged treatment) or left naturally exposed to the rain (Open treatment). Different letters denote significant differences between the Sprayed and Open treatments. Sample size (n) refers to the number of capsules used in each treatment. Test statistics are also shown, Mann-Whitney U, for the number of immobilized seeds and z for the percentage of capsules. * = P < 0.05, ** = P < 0.0001, ns = non-significant differences.

*Tillandsia* species	Number of immobilized seeds							Percentage of capsules with immobilized seeds			
	Sprayed		Open		Bagged						
	n	Mean ±SD	n	Mean ± SD	n	Mean	U	Sprayed	Open	Bagged	z
*T*. *caput-medusae*	10	53.4^a^ ± 47.7	89	5.1^b^ ± 18.6	10	0.0	165**	70^a^	10^b^	0	4.4*
*T*. *achyrostachys*	10	10.6^a^ ± 14.4	86	7.9^b^ ± 26.6	10	0.0	208**	70^a^	16^b^	0	3.5*
*T*. *schiedeana*	10	29.5^a^ ± 38.7	87	5.0^b^ ± 21.6	10	0.0	221*	60^a^	13^b^	0	3.3*
*T*. *circinnatioides*	4	24.5^ns^ ± 39.2	42	18.5 ± 44.8	4	0.0	51^ns^	75^ns^	24	0	1.6^ns^
*T*. *recurvata*	10	10.6^a^ ± 18.4	80	0.8 ^b^ ± 4.8	10	0.0	213**	40^a^	2.5^b^	0	3.8*

#### Seed capture and dispersion distance between hosts and strata

On average, 5.7 ± 4.5 unmarked seeds arrived to each *B*. *copallifera* (pooling the number of seeds between the traps of each tree), while 5.8 ± 7.6 seeds arrived to each *C*. *multiflora*. On comparison of these averages, no difference was found between the numbers of seeds that arrived to either species (*t* = 0.04, *d*.*f*. = 12, *P* = 0.97).

In *Bursera copallifera*, the quantity of captured seeds differed among strata (χ^2^ = 11.4, *d*.*f*. = 2, *P* = 0.003); the highest quantity of seeds was captured on the exterior branches (28 seeds), followed by the interior branches (14 seeds) and finally the trunk (9 seeds). No seeds were captured on the trunk of *C*. *multiflora* and the quantity of seeds captured did not differ between the exterior (13 seeds) and interior (16 seeds) branches (χ^2^ = 0.31, d.f. = 1, *P* = 0.58).

The probability of a trap capturing at least one seed also differed among the strata of *B*. *copallifera* (χ^2^ = 6.75, *d*.*f*. = 2, *P* = 0.03). The highest probability of capturing seeds occurred in the exterior branches (44.4%), which did not differ from the interior branches (27.8%), but did from the trunk (16.7%). The probability of capturing seeds did not differ between the interior branches and trunk. In *C*. *multiflora*, the probability of capturing seeds was similar (*z* = 0.76, P > 0.05) between exterior (15%) and interior (30%) branches.

**Fig 2:**
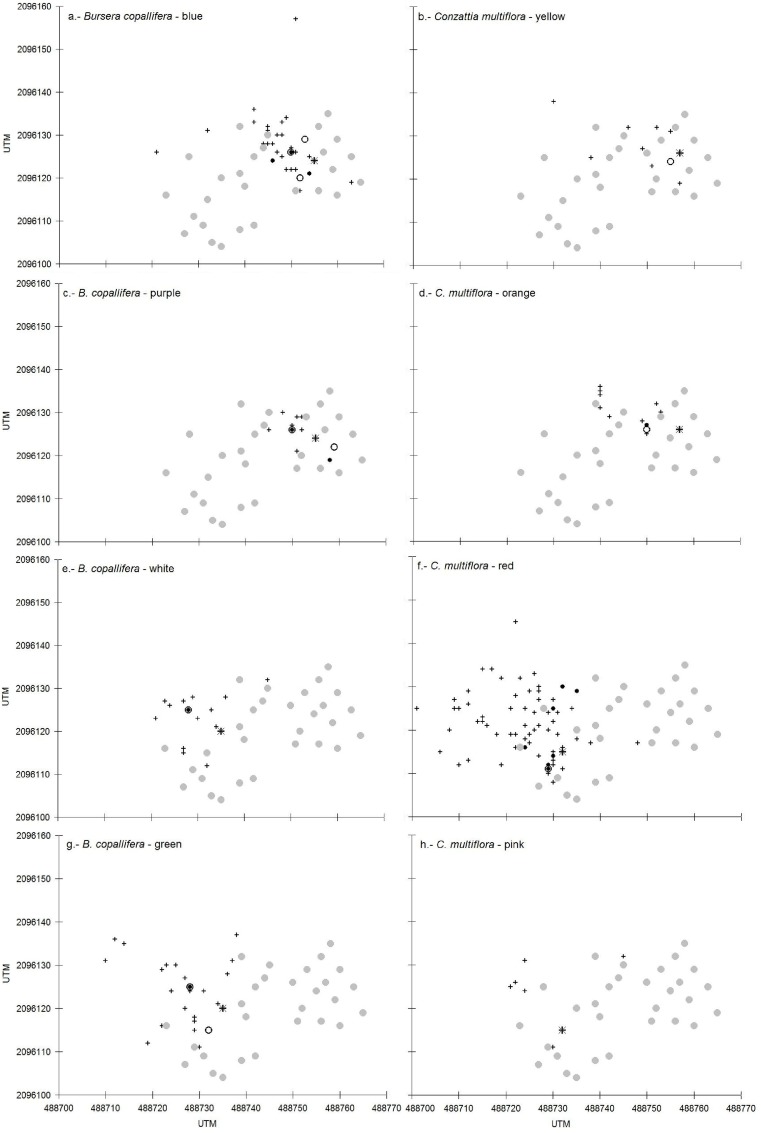
**Fate of seeds experimentally released from the crown of two *Bursera copallifera* (asterisks, a, c, e, g,) and two *Conzattia multiflora* (asterisks, b, d, f, h) trees in the tropical dry forest of San Andrés de la Cal, central Mexico**. The color cited after the species name refers to the color used to paint the seeds in each assay. Painted seeds either remained in the same crown from which they were released (asterisks), arrived at a neighboring tree with seed traps (empty circles), arrived at a shrub or vine without traps (solid small circles), landed on the forest floor (crosses) or did not arrive at a neighboring tree with seed traps (gray circles).

In all of the assays, none of the marked seeds remained in the strainers. The seeds that were released from the crowns of the trees travelled in a northeasterly direction (Fig 2). In each assay, the seeds travelled between 5 and 12 m, with maxima that ranged between 10 to 37 m. With the exception of one assay (*B*. *copallifera*, white powder) (Table 2), most of the seeds (> 60%) could not be found following their release, which suggests that they dispersed beyond the area we searched (our maximum distance was 37 m) or were lost to granivory. Only 8 trees with traps received seeds (Fig 2); the highest quantity of points of collection of seeds was on the forest floor (Fig 2), in six assays <10% fell onto the forest floor, but in two assays, this percentage was greater than 15% (Table 2).

**Table 2:** Number and percentage (in parentheses) of seeds experimentally released from the crown of two *Bursera copallifera* and two *Conzattia multiflora* trees in the tropical dry forest of San Andrés de la Cal, central Mexico. Colors cited refer to the color used to paint the seeds in each assay. Painted seeds remained in the same crown from which they were released (Trapped), arrived at a tree with seed traps, arrived at a shrub or vine without traps, landed on the forest soil or were not found.

Seed fate	*Bursera copallifera*				*Conzattia multiflora*			
	Blue	Purple	White	Green	Yellow	Orange	Red	Pink
Trapped	855 (17.1%)	349 (7.0%)	2444 (48.9%)	803 (16.1%)	25 (0.50%)	738 (14.8%)	4 (00.1%)	143 (2.9%)
Tree with seed traps	221 (4.4%)	16 (0.30%)	1 (0.00%)	4 (0.10%)	206 (4.1%)	71 (1.40%)	3 (00.1%)	0 (0.00%)
Shrub or vine	277 (5.5%)	17 (0.30%)	3 (0.10%)	3 (0.10%)	2 (0.00%)	33 (0.70%)	92 (1.8%)	0 (0.00%)
Forest soil	345 (6.9%)	223 (4.5%)	800 (16.0%)	231 (4.6%)	113 (2.3%)	228 (4.60%)	1708 (34.2%)	391 (7.8%)
Not found	3302 (66.0%)	4395 (87.9%)	1752 (35.0%)	3959 (79.9%)	4654 (93.1%)	3930 (78.6%)	3193 (63.9%)	4466 (89.3%)

In all of the assays, most seeds travelled beyond the crown of the tree from which they were dispersed and only a minority of seeds that remained in the same crown (“trapped”, Table 2). The exception to this was one assay in *B*. *copallifera* (white seeds). The quantity of the seeds that remained in the same tree from which they were released differed between *B*. *copallifera* and *C*. *multiflora* (U = 2.0, P = 0.043); in *B*. *copallifera*, an average of 1113 ± 916 seeds were trapped, while in *C*. *multiflora* an average of 228 ± 346 seeds were trapped. This means that the probability of a seed being trapped was 5 to 1 between *B*. *copallifera* and *C*. *multiflora*.

**Fig 3:**
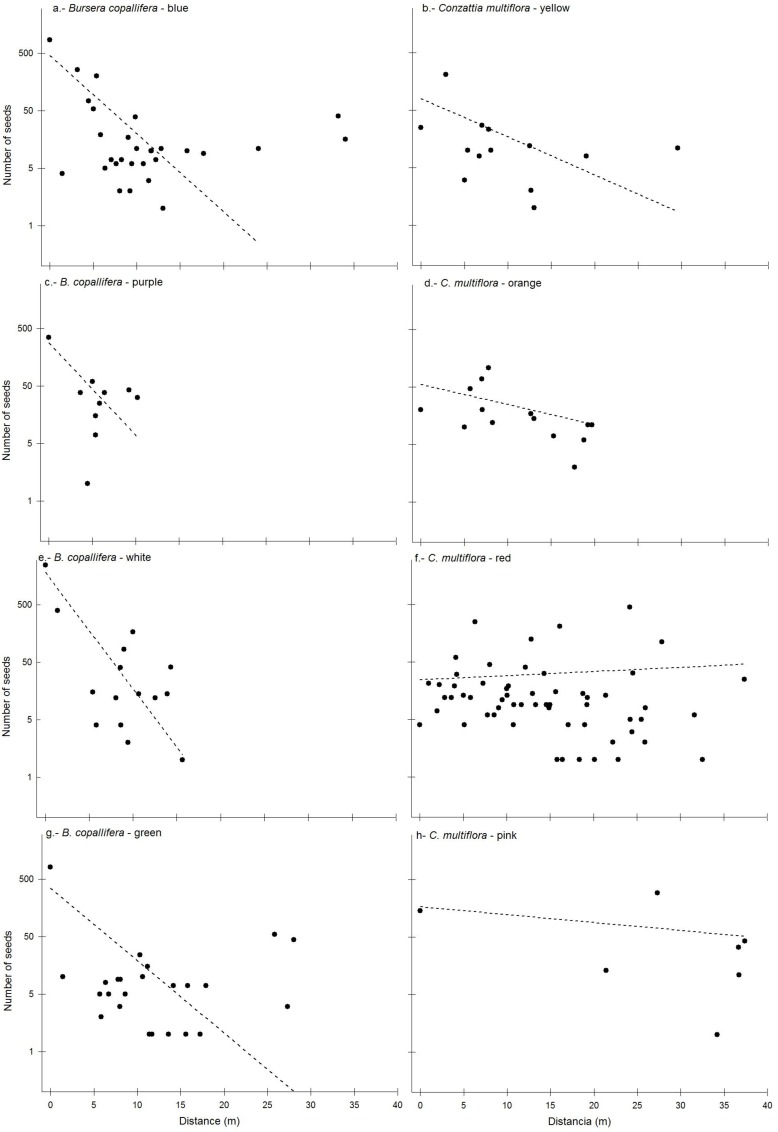
**Relationship between the distance and the number of seeds experimentally released from the crown of two *Bursera copallifera* (a, c, e, g,) and two *Conzattia multiflora* (b, d, f, h) trees in the tropical dry forest of San Andrés de la Cal, central Mexico**. The color cited after the species name refers to the color used to paint the seeds in each assay. Dashed lines correspond to the number of seeds expected according to a generalized linear model with a Poisson distribution.

The generalized linear model shows that there is an effect of tree species (χ^2^ = 2691.8, *P* < 0.0001), distance (χ^2^ = 5278.4, *P* < 0.0001) and of the interaction among tree species, assay and distance (χ^2^ = 719.0, *P* < 0.0001) in the dispersion of seeds (Fig 3). All the curves of dispersion differ among each other (Fig 3), but when the seeds were released from a *Bursera copallifera* (10.2 m ± 6.9 m), they travelled a shorter average distance than when released from *Conzattia multiflora* (14.3 m ± 9.5 m).

### Seed adherence to host branches

For *Tillandsia hubertiana*, the number of seeds captured differed significantly (*t* = 6.4, *d*.*f*. = 9, *P* < 0.001) between hosts. In *B*. *copallifera*, the average number of *T*. *hubertiana* seeds captured was 13.7 ± 3.9, while in *C*. *multiflora* this value was 3.5 ± 2.5 seeds. For *T*. *recurvata*, there were also significant differences (*t* = 6.3, *d*.*f*. = 9, *P* < 0.001) in the number of seeds captured between hosts. In *B*. *copallifera*, the average number of *T*. *recurvata* seeds captured was 13.8 ± 4.9 seeds, while on the branches of *C*. *multiflora*, this value was 2.9 ± 2.1. On average, the branches of *B*. *copallifera* captured 4 times more *T*. *hubertiana* seeds and 5 times more *T*. *recurvata* seeds than those of *C*. *multiflora*.

## Discussion

The dispersion of diaspores among potential habitats is a key process for species maintenance, especially in species with metapopulation dynamics, where the constant establishment of new subpopulations is crucial, considering the turnover of habitable zones [30, 31, 32]. For the epiphytes, the trees and their branches constitute ephemeral habitats and colonization of new trees is a process that is fundamental to the maintenance of their populations [15, 30]. However, trees differ in terms of their quality as hosts [8]. Our results show that the differential capacity of the seeds to adhere to the trees can help to partially explain bias in the distribution of epiphytes among hosts and vertical strata.

It has been suggested [6, 16, 33] that the dispersion period of anemochorous diaspores of the tropical dry forest has been selected to occur during the dry season, when the trees have lost their foliage and high temperatures and dry winds favor both the drying and dispersion of the diaspores. The consequent prediction is that dispersion must occur prior to the rainy season [18]. We found that the phenology of dispersion in *Tillandsia* species followed the pattern expected and was concentrated towards the end of the dry season. The variations we observed between years and between species suggest that the environmental factors that dictate capsule opening vary between years; however, the relatively low number of years observed prevents us from hypothesizing which environmental factor dictates capsule opening. In some species, there might still be selection against late capsule opening; while all of the species dispersed sufficiently prior to the rains, *T*. *circinnatioides* was the latest and presented the highest immobilization of seeds caused by early rains.

It has been hypothesized that epiphyte populations can be influenced by seasonal climatic variation [16], especially the intensity and duration of rainy season. If early, rains could impede dispersion and, if late, they could negatively influence germination and establishment. In agreement with the prediction of these authors, the treatment of moistening seed capsules with water increased both the frequency of capsules with immobilized seeds and the number of these seeds, except for *T*. *circinnatioides*. In capsules isolated from the rain, however, there were no immobilized seeds. Immobilization of seeds by early rains caused losses ranging between 2.5 and 24% of the fruits, reaching a maximum in the species that dispersed closest to the rainy season (*T*. *circinnatioides*). Immobilization of seeds represents a direct loss, because immobilized seeds remain attached to the inflorescence and, even if they do germinate, they fall to the forest floor along with the decaying inflorescence.

Previous studies in the tropical dry forest of San Andrés de la Cal show that *Tillandsia* species are vertically stratified [10]. The results of this study partially explain the distribution of epiphytes among strata, since the natural capture of seeds was concentrated in the exterior strata, while little seed capture took place on the trunk. It was observed during the dispersion experiment that the seeds did not simply fall, but rather floated with the wind, causing dispersion distance to be greater with increased height of the seed source (*e*.*g*. *C*. *multiflora*) [15, 16]. Epiphytes in the upper stratum (or in taller trees such as *C*. *multiflora*) produce seeds that travel further and do not necessarily provide seeds to the trunk, while epiphytes in the lower strata do provide seeds to the upper strata. The lack of seeds on the trunk can therefore be explained by the fact that: a) trunks constitute the largest structures but also the scarcest [10]; b) in general, seeds dispersed by the wind collide less with trunks than with branches [34]; c) *Tillandsia* seeds rise and do not simply fall, and d) the seed capture network formed by the branches impedes the seeds from reaching the trunk. This strongly supports the notion [10, 21] that the higher quantity of supports (structures) in the exterior part of the crown form a seed capture network. However, we did not observe seeds reaching the trunks of *C*. *multiflora* and a previous study suggests that epiphytes are more numerous on the trunk of this host [10]. The open crowns of this host increase the probability of seeds reaching the trunk, but the low number of *C*. *multiflora* trees observed possibly prevented us from observing the arrival of seeds.

We did not find differences in the number of unmarked seeds captured in the traps between *B*. *copallifera* and *C*. *multiflora*; this shows that seeds arrive to both hosts in the same number but that their establishment is only possible if sufficient adhesion to the bark can be achieved. While the morphological characteristics of the bark are not linked to the host quality in our site of study [11], the data of marked seeds and those from the wind duct experiment showed that seed capture differs between tree species. We hypothesized that there would be high seed capture in the host trees that naturally concentrate species of *Tillandsia*. The data show that the seeds produced by epiphytes on *B*. *copallifera* are trapped within its crown; however, even with higher seed capture, *B*. *copallifera* generally exports more than half of the seeds produced in its crown. Based on this finding, a *Tillandsia* plant that colonizes a *B*. *copallifera* individual has a high probability of colonizing neighboring branches with its own seeds; while a *Tillandsia* that colonizes a *C*. *multiflora* will export most of its seeds. In addition to bark adherence, two factors that must favor the trapping of seeds within *B*. c*opallifera* are the resins that are usually present in its bark and its density of branches. While *C*. *multiflora* has an open and less dense crown, the crown of *B*. *copallifera* is denser [10]. This greater density of branches in *B*. *copallifera* could reduce wind speed, generate turbulence and increase the number of collisions between seeds and branches [34]. Such modifications to wind speed and direction, added to the adherence of the bark, could explain both the high seed capture values inside the crown and the high epiphyte concentration presented in this host.

Colonization over long distances has been inferred for many epiphyte species due to their distribution in patches, and it is a characteristic that has been associated with a mechanism of speciation in these plants [35] as well as with metapopulational dynamics within the forest [31, 32]. In the study zone, some seeds of *Tillandsia* that leave the crown, can travel distances greater than 37 m. Interestingly, the marked seeds did not massively colonize the trees that surround the source trees and dispersion was found to present a bias in a northeasterly direction. This shows that dispersion can present a directional bias and that the trees to the east of the seed sources would have a higher probability of being colonized. This directional bias is a clear consequence of the season of dispersion, during which the predominant winds travel in an easterly or northeasterly direction [22].

In the tropical dry forest of Morelos, in Mexico, *C*. *multiflora* is abundant and is an indicator species of forest maturity [25]; however, it is considered a limiting host for epiphytes [11]. The data suggest that this limitation occurs because it does not capture the seeds of the *Tillandsia* species that reach it. In contrast, *B*. *copallifera* and other species of *Bursera* are hosts that concentrate the epiphyte species [11]. This concentration is the result of both the capture of seeds that allows the host trees to be colonized by seeds that are external to their crowns and the trapping of seeds when the original colonizing epiphytes reproduce.

## Conclusions

Our experimental data show that factors that limit dispersion are direct causes that help to explain the biased patterns of distribution in the epiphytic species. Our data also show that *Tillandsia* species that have not been selected to synchronize their dispersion periods with the rain show little abundance, suggesting that these species cannot colonize new habitats, while those that have dispersion periods that are synchronized with the rains can colonize favorable habitats in which subsequent recolonization should generate abundant populations that can in turn function as sources for the colonization of new habitats.

## Supporting information

S1 Fig Immobilized seeds or seedlings that originated from these seeds. *Tillandsia achyrostachys* E. Morren ex Baker (a), *T*. *caput*-*medusae* E. Morren (b), *T*. *hubertiana* Matuda (c), *T*. *makoyana* Baker (d), *T*. *recurvata* (L.) L. (e) and *T*. *schiedeana* Steud. (f). All of the images were recorded in the tropical dry forest of San Andres de la Cal, in central Mexico.
(TIF)

S2 Fig A seed trap attached to a branch of *Bursera copallifera*. (TIF)

S3 Fig Glass duct used to test the adherence of *Tillandsia* seeds. (TIF)
