# Supplementary figures and images for: Dispersal limitation of Tillandsia species correlates with rain and host structure in a central Mexican tropical dry forest
Source: PLoS One. 2017 Feb 3;12(2):e0171614. doi: 10.1371/journal.pone.0171614 (PMC5291420; doi:10.1371/journal.pone.0171614)

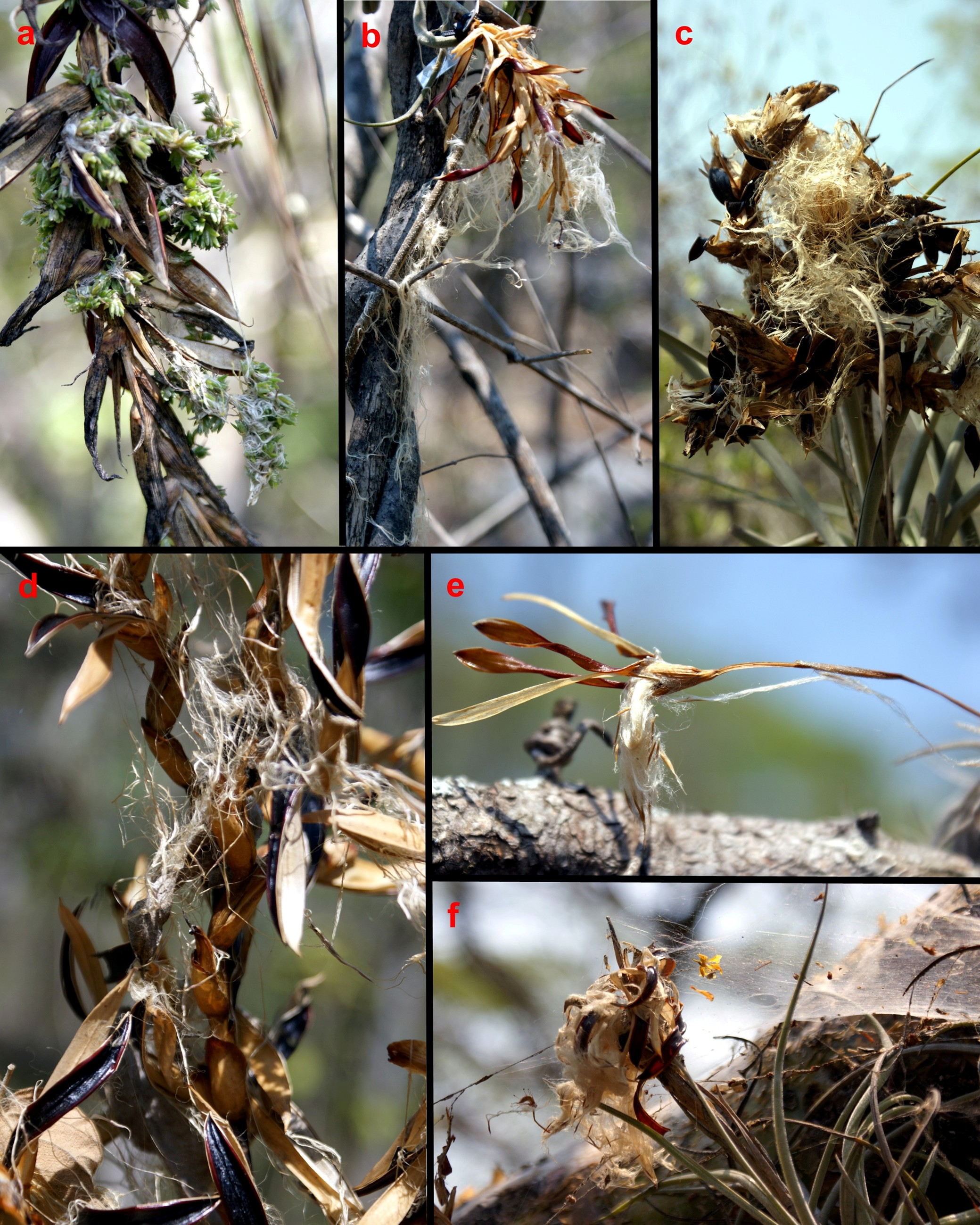

Supplement: S1 Fig — Tillandsia achyrostachys E. Morren ex Baker (a), T. caput-medusae E. Morren (b), T. hubertiana Matuda (c), T. makoyana Baker (d), T. recurvata (L.) L. (e) and T. schiedeana Steud. (f). All of the images were recorded in the tropical dry forest of San Andres de la Cal, in central Mexico. (TIF) [file pone.0171614.s001.tif]

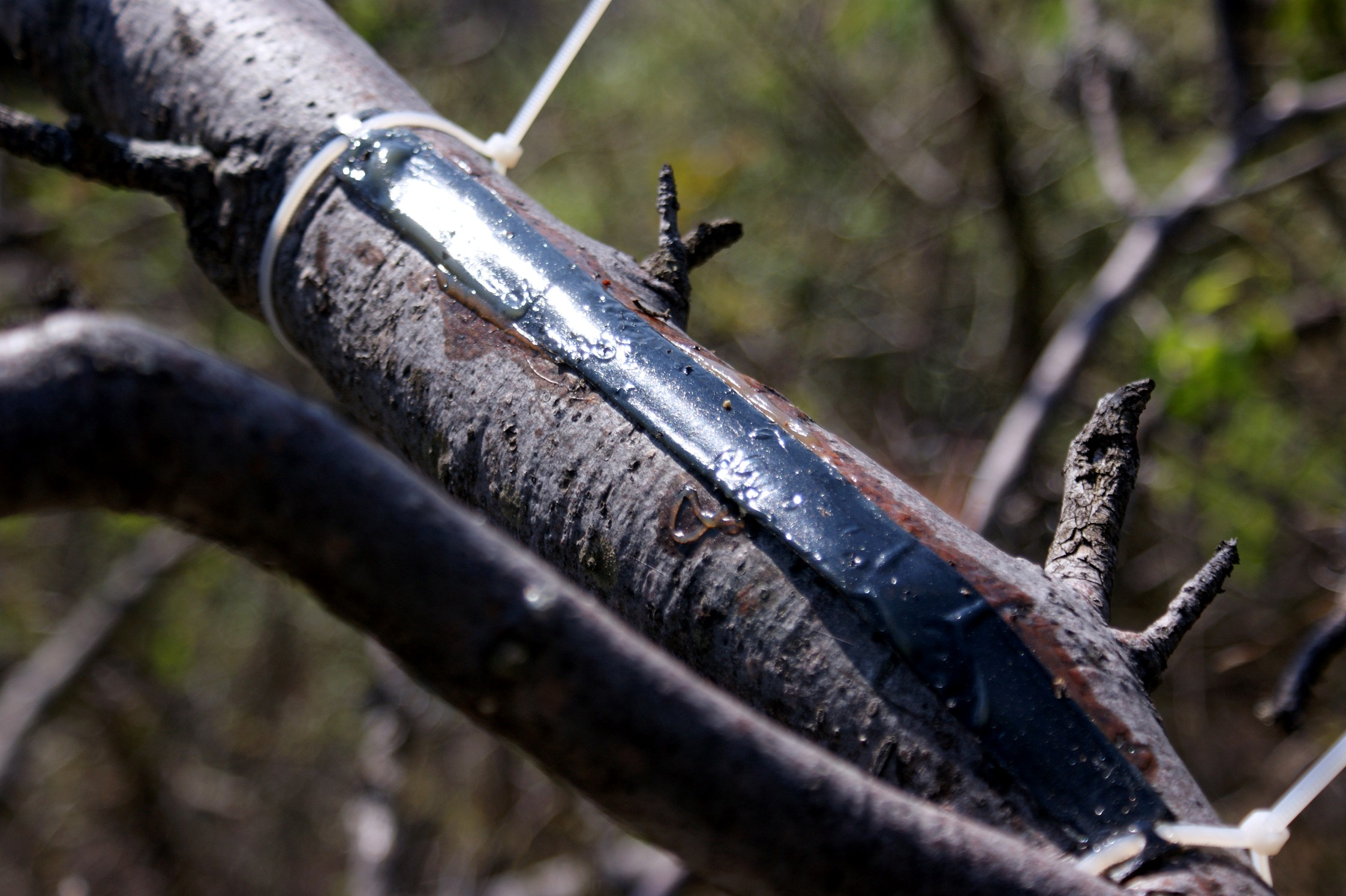

Supplement: S2 Fig — (TIF) [file pone.0171614.s002.tif]

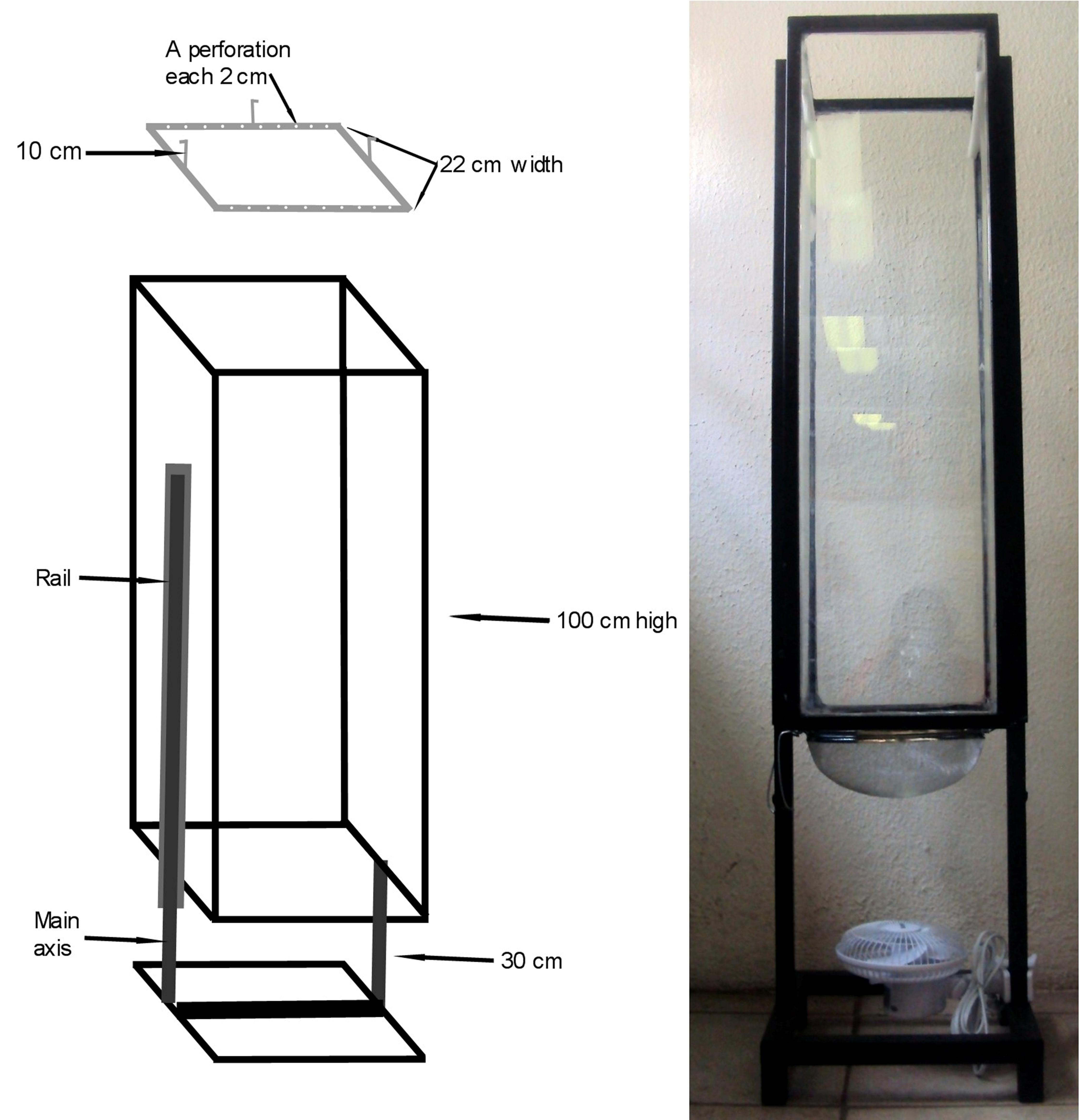

Supplement: S3 Fig — (TIF) [file pone.0171614.s003.tif]
